# Sarcopenic obesity: epidemiology, pathophysiology, cardiovascular disease, mortality, and management

**DOI:** 10.3389/fendo.2023.1185221

**Published:** 2023-06-30

**Authors:** Shibo Wei, Thanh T. Nguyen, Yan Zhang, Dongryeol Ryu, Karim Gariani

**Affiliations:** ^1^ Department of Molecular Cell Biology, Sungkyunkwan University School of Medicine, Suwon, Republic of Korea; ^2^ Department of Biomedical Science and Engineering, Gwangju Institute of Science and Technology (GIST), Gwangju, Republic of Korea; ^3^ Division of Endocrinology, Diabetes, Nutrition and Therapeutic Patient Education, Department of Medical Specialties, Geneva University Hospitals, Geneva, Switzerland; ^4^ Diabetes Center, Faculty of Medicine, University of Geneva, Geneva, Switzerland

**Keywords:** sarcopenia, obesity, pathogenesis, endocrine, cardiovascular disease

## Abstract

Sarcopenic obesity is defined as the coexistence of sarcopenia and obesity in the same individual, characterized by of the co-presence of body fat accumulation and muscle loss. This condition is currently a major concern as it is associated with frailty and disabilities such as cardiovascular disease, fractures, dementia, cancer, and increased all-cause mortality. Particularly, older individuals remain at risk of sarcopenic obesity. Progress at several levels is needed to improve the global prognostic outlook for this condition, including the elaboration and implementation of a more uniform definition that may favor the identification and specification of prevalence by age group. Furthermore, improvements in the understanding of the pathogenesis of sarcopenic obesity may lead to the development of more specific therapeutic interventions to improve prognosis. We reviewed the knowledge on sarcopenic obesity and its associations with cardiovascular diseases and mortality.

## 1 Introduction

Sarcopenic obesity is defined as a functional and clinical condition characterized by the coexistence of loss of skeletal muscle mass and function and an excess of adipose tissue. The incidence of sarcopenic obesity is increasing rapidly, mainly owing to the aging of the worldwide population and the current obesity epidemic. Sarcopenic obesity is associated with several clinical complications such as frailty, fractures, cardiovascular diseases, cancer, and an increased risk of hospitalization and mortality. Physiological aging may be associated with a modification in body composition, including redistribution of adipose tissue, and it is characterized by a decline in muscle mass, which can lead to the development of sarcopenic obesity. Sarcopenia and obesity are distinct entities, but they share common pathophysiological features and risk factors such as lifestyle, aging, production of inflammatory cytokines and reactive oxygen species, and endocrine alterations. Moreover, the two conditions act synergistically to enhance each other, leading to a detrimental vicious circle. The absence of globally adopted common definition criteria for sarcopenic obesity impairs precise epidemiological assessment, proper patient identification, and, ultimately adapted management to limit related adverse complications. In this review, we discuss the definition, epidemiological data, and pathogenesis of sarcopenic obesity, in addition to the associated cardiovascular diseases, mortality-related complications, and management.

## 2 Sarcopenia and obesity

Sarcopenia is defined as the presence of progressive and generalized loss of muscle mass and strength associated with harmful outcomes such as falls, fractures, frailty, and mortality. Sarcopenia more frequently affects older individuals, but the progressive decrease in muscle mass begins once an individual reaches their 40s, and after the age of 50 years, muscle mass decreases yearly by 1%–2% (1).

The mechanisms that trigger sarcopenia are complex and include a decline in neural function with a reduction in motor units and fibers, hormonal changes, reduced satellite cell function, malnutrition, chronic low-grade inflammation, mitochondrial dysfunction, and behavioral factors such as a sedentary lifestyle. Therefore, simple and unique diagnostic criteria have not yet been established to define sarcopenia. Various definitions have been proposed for diagnosing sarcopenia, most of which use a set of criteria based on muscle strength, mass, and gait speed (2).

In 2019, the European Working Group on Sarcopenia in Older People (EWGSOP) published the updated EWGSOP2 guidelines that focused on low muscle strength using grip strength as a central factor for diagnosing sarcopenia, as muscle strength has been shown to better predict poor outcomes than muscle mass in sarcopenia (3–5). Reduced muscle quality and quantity (DXA or alternative tests such as mid-thigh imaging by MRI or psoas muscle measurement with CT) were used to confirm sarcopenia, and poor physical performance was assessed using the Timed Up and Go test or gait speed to determine its severity (3).

Due to the absence of a standardized and homogeneous definition of sarcopenia, it remains difficult to precisely establish its prevalence. A meta-analysis of 151 studies performed in countries from each continent, and comprising a total of 692 056 individuals with a mean age of 68.5 years, found that the prevalence of sarcopenia ranged from 10% to 27% (6).

Altogether, the precise prevalence of sarcopenia remains unclear as it varies markedly according to the classification and cut-off points used. Future studies assessing the prevalence of sarcopenia should adhere to the current guidelines, thereby allowing for comparison between regions and easier global estimation of the prevalence of this disorder.

The World Health Organization (WHO) defines overweight and obesity as abnormal or excessive fat accumulation that may impair health, with BMIs of 25.0 to 29.9 kg/m^2^ and BMI ≥ 30.0 kg/m^2^ for overweight and obesity. Obesity is a chronic disease with an increasing prevalence that has reached pandemic proportion over the past several decades. The prevalence of overweight and obesity has more than doubled worldwide since 1980, and approximately 30% of the global population is overweight or obese. The prevalence of obesity has increased in both sexes over all age groups, but this increase has been proportionally higher in older individuals and women (7). Obesity represents a major health challenge as it can increase various disorders such as type 2 diabetes, cardiovascular diseases, cancer, dementia, and depression, which can all lead to a significant reduction in the quality of life or even lead to premature death. Obesity has replaced smoking as the foremost lifestyle-related risk factor for premature death and is associated with a significant increase in healthcare costs by up to 30% compared to that in individuals with normal BMIs (8).

The concept of the “obesity paradox” may apply specifically to older population; therefore, the impact of obesity on cardiovascular disease and mortality remain unclear (9, 10). A meta-analysis including approximately 200 000 individuals aged ≥ 65 years showed that being overweight was not associated with an increased risk of mortality and that BMIs lower than 23 kg/m^2^ and higher than 33 kg/m^2^ were associated with an increased risk of mortality, suggesting a U-shaped curve of BMI for mortality (11). A potential explanation for the

“obesity paradox” is the approximate estimation of body fat by BMI;, body composition is not assessed by this index, including the distinction between lean and fat body mass, which opposite impacts on the risk of mortality. An increased risk is associated with fat mass and, and the mortality risk is negatively associated with muscle mass (12).

## 3 Sarcopenic obesity

The available definitions of sarcopenic obesity use individual definitions of sarcopenia and obesity. There is a lack of a globally accepted classification of sarcopenia and, therefore, sarcopenic obesity. Owing to the lack of consensus on the cut-off points for sarcopenia and obesity, standard diagnostic criteria for sarcopenic obesity are yet to be established. Consequently, the epidemiological estimation of sarcopenic obesity remains imprecise, and, its prevalence varies according to the definition used. A prospective cohort called South Korea’s Sarcopenic Obesity Study comprising healthy volunteers aged 20-80 years of age found a prevalence of sarcopenic obesity ranging from 0.8% to 22.3% in women and from 1.3% to 15.4% in men (13). Data from individuals aged 18–90 years from the Dutch Lifelines cohort study showed a global prevalence of sarcopenic obesity of 1.4% and 0.9% in women and men, respectively, with a rise in prevalence at 50 years of age and a prevalence reaching 16.7% in the 80-89 years age group (14). A meta-analysis including 50 studies representing 86 285 people found a global prevalence of 11% of sarcopenic obesity in adults aged 60 years of (15).

Sarcopenic obesity appears to be a more severe condition than obesity alone, as it is associated with a higher risk of several disorders such as cardiovascular disease, reduced bone mineral density, and all-cause mortality (16–18).

## 4 Pathogenesis of sarcopenic obesity

**Figure 1:**
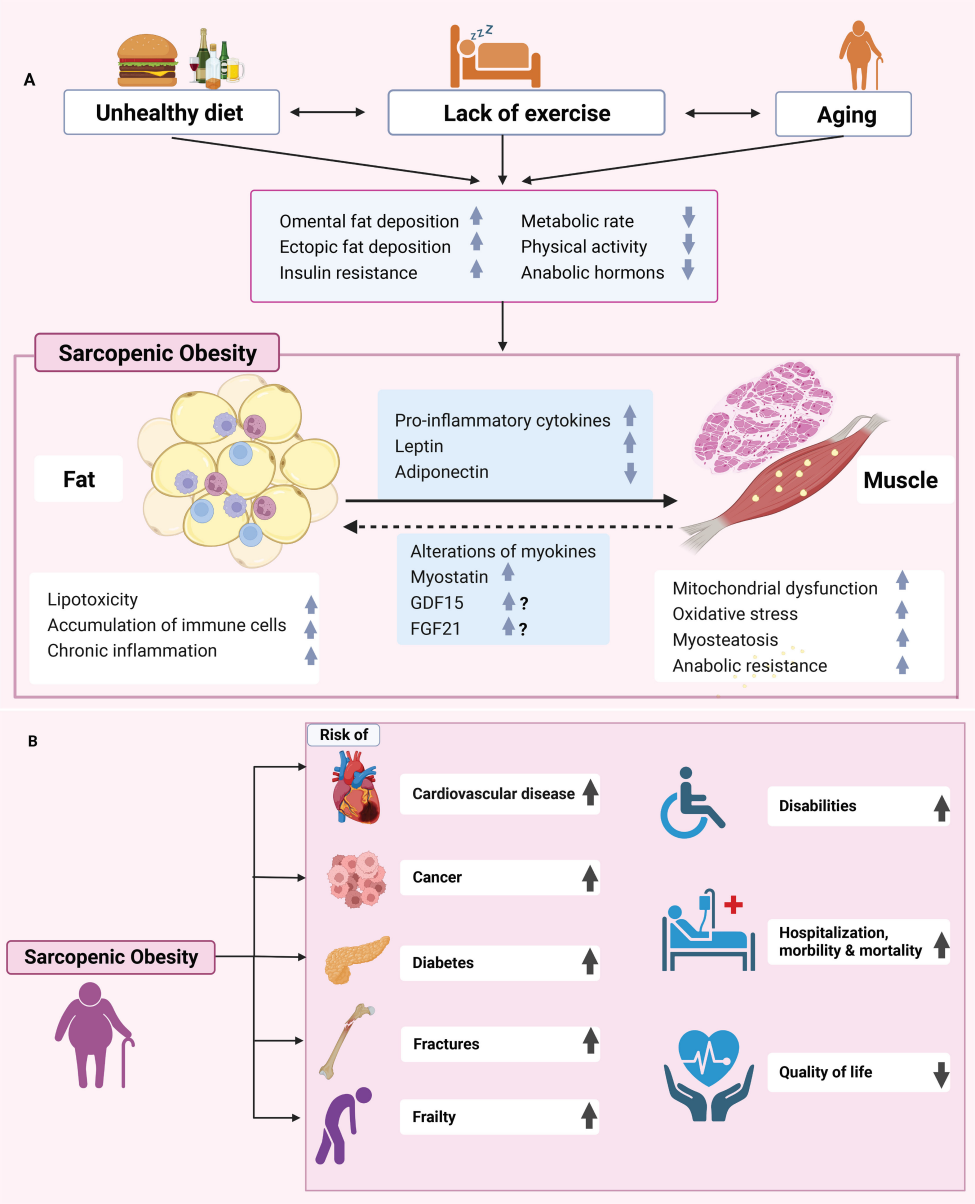
**(A)** Pathophysiology and risk factors for sarcopenic obesity development. Aging and obesity that is induced by unhealthy diet and lack of exercise will promote the development of sarcopenic obesity. Sarcopenic obesity is associated with several deleterious biological mechanisms such as insulin resistance, lipotoxicity, mitochondrial dysfunction, oxidative stress, chronic inflammation, and proteostasis. Sarcopenic obesity is characterized by adipose tissue expansion and muscle loss that cause increased pro-inflammatory cytokine levels, increased leptin, reduced adiponectin, and intramyocellular lipid deposits. Individuals with sarcopenic obesity are more at risk of several conditions such as cardiovascular disease, cancer, diabetes, fracture, and hospitalization. GDF15, Growth differentiation factor 15; FGF21, Fibroblast growth factor 21. **(B)** As a result of sarcopenic obesity, there is an increased risk of cardiovascular disease, cancer, diabetes, fractures, and frailty, as well as hospitalizations, morbidity and mortality, disability, and decreased quality of life.

The pathogenesis of sarcopenic obesity remains complex, multifactorial, and only partially elucidated. Low-grade inflammation has a substantial role in the pathogenesis of sarcopenic obesity, and both obesity and sarcopenia have significant roles in this (**Figure 1**). The expansion of white adipose tissue promotes the secretion of proinflammatory cytokines that facilitate the accumulation of inflammatory cells such as macrophages, inflammatory T lymphocytes, and mast cells in not only adipose tissue but

also muscle tissue. In the context of obesity, adipose tissue macrophages (ATM) change their phenotype from M2 to M1. M2 or alternatively activated macrophages induced adipose tissue to exhibit anti-inflammatory properties (19). In contrast, M1 ATM secrete proinflammatory molecules, including IL-6, TNF-α, IL-1β, MCP-1, CCR2, and CCR5 (20). The same phenomenon is observed in muscle tissues, as cytokines produced by infiltrating cells such as macrophages can cause muscular atrophy by inducing filament protein degradation and apoptosis (21). Furthermore, sarcopenic obesity is often characterized by intramuscular adipose tissue and the intramyocellular accumulation of lipids composed of triacylglycerol, ceramide, and diacylglycerol, which are characteristic of lipotoxicity mechanisms. The depositions of lipids and their metabolites in muscle tissue is also called myosteatosis. These lipids can induce a reduction of GLUT4 translocation via impairment of insulin signaling, thereby leading to a reduction of glucose uptake by skeletal muscle. As a consequence, fatty acid oxidation increases in the mitochondria, causing a concomitant increase in the ATP/ADP ratio and decrease in electron transport chain that impairs oxidative phosphorylation, thus promoting ROS production, inflammation, and ultimately sarcopenia (22). Several mechanisms linking insulin resistance to loss of muscle mass and function have been proposed. Insulin and amino acids together promote protein synthesis and, therefore, increase muscle mass. The protein complex, mTORC1, seems to play a central role in this process because amino acids and insulin act synergistically to activate it, which itself plays a central role in the insulin signaling pathway (23). In mice, the deletion of mTORC1 leads to high muscle atrophy, and in humans, the daily ingestion of 10 g of essential amino acids induces a 60% increase in muscle protein synthesis levels that is absent when mTORC1 inhibitors are simultaneously administered, confirming that mTORC1 is a key regulator of protein synthesis (24, 25). At the molecular level, insulin alleviates the inhibitory effect of the TSC complex on mTORC1 by promoting AKT activation, whereas amino acids can directly increase the activation level of mTORC1. Increased mTORC1 activation induces the formation of muscle cells proteins (26, 27).

The FoxO family is another important contributor linking insulin resistance with muscle atrophy. The FoxO family, which is indirectly inhibited by insulin, acts mainly by regulating protein catabolism through the activation of the proteolytic system. Therefore, the reduced action of insulin in the insulin-resistant state may promote protein degradation of the FoxO family through caspase-3 and the ubiquitin–proteasome proteolytic pathway, ultimately leading to sarcopenia (28, 29).

Altogether, there is a body of evidence indicating that insulin resistance induces muscle wasting through mTORC1 and Foxo; however, further studies are required to fully elucidate the induction of sarcopenia by insulin resistance.

Sarcopenic obesity is associated with an imbalance between the antioxidant system and oxidative entities, leading to an accumulation of ROS characteristic of oxidative stress. ROS alter mitochondrial function and mass, increase mitochondrial DNA damage, reduce ATP synthesis, and elicit apoptotic pathway activity, which altogether, lead to a reduction in muscle mass. Oxidative stress can also lead to ER stress, which is characterized by unfolded protein response (UPR) causing dysfunction of proteasomal degradation and autophagy, which reduces protein levels and causes an imbalance in calcium homeostasis that altogether favor a decrease in muscle mass (30, 31).

Age-related endocrine changes involve various hormones and may play a role in the development and progression of sarcopenic obesity. Testosterone is an anabolic hormone that promotes protein synthesis in the skeletal muscle cells. In men, aging is associated with a decline in testosterone levels, and obesity itself induces the conversion of testosterone to estradiol through the aromatization process that takes place in the adipose tissue. Thus, hypogonadism leads to sarcopenia (32).

In postmenopausal women, the reduction in estradiol and increase in FSH levels promote body composition changes comprising an increase in adipose tissue and a decrease in muscle mass. In addition, the levels of growth hormone (GH), another anabolic hormone involved in muscle mass through the PI3K-AKT/PKB-mTOR pathway, is reduced with aging and obesity and participates in the pathogenesis of sarcopenic obesity (33).

Growth differentiation factor 15 (GDF15) has been implicated in aging, inflammation, mitochondrial dysfunction, cachexia, and sarcopenia (34, 35). It is likely that these changes are also altered in patients with sarcopenic obesity; however, this needs to be confirmed. Fibroblast growth factor 21 (FGF21), which is similar to GDF15, is closely associated with mitochondrial dysfunction and protects against obesity and fatty liver disease by acting on brown fat. Increased levels have also been observed in patients with sarcopenia (36). This has not yet been confirmed in patients with sarcopenic obesity but is certainly a possibility. The expression and secretion of many other myokines may be altered in sarcopenic obesity, and this may participate directly or indirectly in muscle wasting, obesity, and metabolic disorders (**Figure 1**).

Notably, sarcopenic obesity and lean sarcopenia are two different clinical entities with relatively similar pathophysiological mechanisms. Comparative pathophysiological studies are lacking; however, it can be hypothesized that the contribution and intensity of different mechanisms, such as insulin resistance, low-grade inflammation, and oxidative stress, differ in the development of sarcopenic obesity followed by lean sarcopenia.

Overall, in sarcopenic obesity, adipose and muscle tissues enter a crosstalk that leads to a vicious circle. Indeed, the expansion of white adipose tissue mass and the local infiltration of muscle tissue by lipid deposits leads to secretion of proinflammatory adipokines, thereby inhibiting protein synthesis and inducing protein catabolism, which exacerbate the decline in muscle mass and t promotes pathological mechanisms such as insulin resistance. Furthermore, the secretion of cytokines by fat mass not only affects muscle tissue but also other organs, such as the liver and white adipose tissue, thereby impairing insulin signaling and leading to insulin resistance, cardiovascular diseases, and ultimately an increased risk of mortality (37, 38).

## 5 Sarcopenic obesity and cardiovascular diseases

Cardiovascular disease (CVD), the major cause of death in Western countries, has recently been linked to sarcopenic obesity, as evidenced by multiple cross-sectional studies (39–41). Sarcopenic obesity significantly increases the risk of cardiovascular risk factors including insulin resistance (IR), metabolic syndrome, and adverse glycolipid metabolism (16, 42–44), even in the context of divergent comorbidities such as cancer and peritoneal dialysis (45, 46). This relationship extends to the point of directly influencing the all-cause mortality of patients after cardiovascular surgery (47). Recent cross-sectional and cohort studies suggest that sarcopenic obesity, as measured by muscle strength rather than muscle mass, is associated with increased risks of CVD (48) and cardiovascular mortality (39); however, this finding is challenged by certain studies that imply that sarcopenic obesity may have weak links to CVD (39, 49) or even no association with CVD, independent of sarcopenia or obesity (41). The inherent limitations of cross-sectional or cohort study designs render it difficult to establish causality, and the direct molecular mechanisms underpinning this association remain poorly understood; hence, more in-depth research is needed to fully elucidate this association. However, it could still, in a sense, be extrapolated indirectly through cardiovascular risk factors.

Atherosclerosis, identified as a root pathological contributor to multiple CVDs, is inextricably intertwined with sarcopenic obesity and is primarily driven by the impact of sarcopenic obesity on metabolic health (50–53). This state is characterized by insulin resistance (IR), which induces vasodilation lesion and endothelial dysfunction, thus fueling the advancement of atherosclerosis (54). In addition, the proinflammatory status, exacerbated by macrophages infiltrating adipose tissue during the progression of sarcopenic obesity, is another causative factor of atherosclerosis (55). These macrophages, in combination with adipocytes and lipid-infiltrated muscle tissues, stimulate the secretion of proinflammatory cytokines, particularly TNF-α and IL-6, while reducing the release of protective adiponectin, thereby evoking systemic chronic inflammation that directly damages the vascular endothelium and, in, turn exacerbates IR and atherosclerosis (56). Sarcopenic obesity-induced adipokine overexpression can also trigger oxidative stress, forcing low-density lipoprotein oxidation (57), cholesterol efflux obstruction, and collagen fiber aggregation in fibroatheroma plaques (58), which, taken together, further deteriorate endothelial dysfunction and aggravate atherogenesis, ultimately provoking multifactorial CVD events, including ischemic heart disease, arrhythmia, and heart failure. Recent research has revealed an independent correlation between sarcopenic obesity and coronary artery calcification (50), which is typically concomitant with advanced atherosclerosis and has been established as a predictor of impending cardiac events (59). The underlying mechanisms may be attributed to the overproduction of ROS, followed by endothelial dysfunction, arterial stiffness, and microvascular damage, highlighting the robust association between sarcopenic obesity and atherosclerosis, and, thereby, CVDs.

Myocardial fibrosis, another well-established cardiac ailment and pathological hazard for a multitude of CVDs, is closely associated with sarcopenic obesity. This is substantiated by robust evidence indicating the roles of overexpressed TNF-α and chronic oxidative stress. These factors stimulate myofibroblasts and activate profibrotic signaling pathways, respectively, thereby promoting collagen production and inducing cardiac fibrosis (55). Additionally, the pathological process of sarcopenic obesity may trigger hyperinsulinemia via IR, which drives mTOR-S6K1 signaling (60) and the TGF-β1-SMAD pathway (61) by activating the renin–angiotensin–aldosterone system, eventually leading to activated myocardial fibrosis in conjunction with the inflammatory status.

Heart failure is closely linked to sarcopenic obesity, as the main plausible explanation is that myocardial fibrosis results in cardiac remodeling and stiffness, which culminates in cardiac hypertrophy and, eventually, heart failure. Moreover, the infiltration of adipose tissue in muscle induces peri-muscular lipid deposition, defined as lipotoxicity, expediting inflammatory cytokine secretion and NF-κB activation for proteolysis and apoptosis, thereby exacerbating muscle fiber damage and, ultimately, heart failure (62), with either a reduced or preserved ejection fraction. Similarly, oxidative stress may also cause mitochondrial malfunction, resulting in mitochondrial DNA alteration and anomalies in the electron transport system. This further aggravates the oxidative injury to the myocardium, ultimately leading to secondary chronic heart failure.

Several cross-sectional studies have indicated that sarcopenic obesity might be independently linked to other CVDs such as myocardial infarction, arrhythmia, and diastolic dysfunction (63, 64). However, these studies lacked explicit causality or mechanistic evidence, indicating the need for further research. Conversely, multiple CVDs accompanied by impaired endothelial function may obstruct blood flow and oxygen diffusion to the skeletal muscles, thus playing a determining role in exercise intolerance and the pathogenesis of sarcopenic obesity (65). In addition, disruption of energy metabolism in individuals with CVD commonly coexists with mitochondrial dysfunction, contributing to decreased oxidative capacity and augmented fat accumulation in skeletal muscles, thus worsening sarcopenic obesity. Notably, individuals with CVDs are prone to experiencing symptom-induced physical activity limitations, such as shortness of breath and fatigue. These restrictions may result in muscle atrophy and decline in physical function, culminating in sarcopenic obesity. Subsequently, sarcopenic obesity may promote a sedentary lifestyle that has been linked to various complications including diabetes, hypertension, and dyslipidemia, all of which are recognized risk factors for CVD. Sedentary behavior amplifies chronic low-grade inflammation and oxidative stress, thereby causing a dual detrimental effect on both CVD and sarcopenic obesity.

Sarcopenic obesity and CVD are mutually causative and intricately interconnected. Further in-depth studies to uncover the underlying mechanisms will provide a deeper understanding of the complex interplay between these pathologies and pave the way for innovative clinical therapies.

## 6 Mortality related to sarcopenic obesity

As both obesity and sarcopenia are associated with the elevated risk of all-cause mortality, the coexistence of both conditions may exacerbate the risk of mortality (**Figure 1**) (66, 67). Multiple prospective studies have assessed the association between sarcopenic obesity and mortality risk. The third National Health and Nutrition Examination Survey (NHANES III) assessed the risk of all-causes mortality in 4652 individuals aged ≥ 60 years, with a follow-up of 14 years. After adjusting for age, sex, cardiovascular risk factors, and ethnicity, a significantly higher risk of all-cause mortality was observed among women with sarcopenic obesity compared to women without obesity or sarcopenia (**Table 1**). However, no significant difference was observed in terms of the mortality risk between men with and without sarcopenic obesity (68). The British Regional Heart Study examined the risk of all-cause mortality among 4107 men aged between 60-79 years. Over a follow-up period of 11 years, men with sarcopenic obesity had the highest risk of mortality compared to control subjects without obesity and without sarcopenia (41). A Swedish prospective study including 809 individuals assessed the risk of mortality with sarcopenic obesity. Women aged  75 years with sarcopenic obesity had a higher risk of 10-year mortality compared to those without sarcopenia or obesity. Among men aged  75 years, a similar association with mortality was observed, although it did not reach statistical significance (69). A cohort study using the UK Biobank (n=452 931) showed a significantly increased mortality risk in sarcopenic obesity om individuals compared to control subjects with previous cardiovascular disease (39). Finally, a meta-analysis of 23 studies including 50 866 individuals showed that sarcopenic obesity was significantly associated with a higher risk of all-cause mortality. Several subgroup analyses have shown that this higher risk of mortality is significant among community-dwelling adults and hospitalized patients. In addition, this significance persisted in studies that used different criteria to define obesity and sarcopenia (18).

**Table 1:** Results of main studies assessing the mortality risk associated with sarcopenic obesity.

Cohort study	Number of individuals	Definition for sarcopenic obesity	Outcome assessed	Comparison	Results
NHANES III (68)	4652	Sarcopenic obesity was defined according to body fat and skeletal muscle mass measured by BIA	all-risk mortality	Women with sarcopenic obesity compared to women without obesity or sarcopenia	HR: 1.29, 95% CI: 1.03-1.60)
British Regional Heart Study (41)	4252	Baseline MAMC and WC measurements were used to classify the participants	all-risk mortality	Men with sarcopenic obesity compared to control subjects without obesity and sarcopenia	HR: 1.72, 95% CI: 1.35–2.18
The Gothenburg H70 Birth Cohort and the Uppsala Longitudinal Study of Adult Men (69)	809	Obesity was defined by any of 3 established definitions: WC 88 cm/ 102 cm for women and men, BMI > 30 kg/m^2^ or fat mass > 30% for men and > 42% for women, and sarcopenia was defined using EWGSOP2 definition.	all-risk mortality	Women 75 years of age with sarcopenic compared to those without sarcopenia or obesity.	HR 3.25, 95% CI (1.2–8.9)
UK Biobank (39)	452 931	Sarcopenic obesity defined by a BMI >30 kg/m^2^ and sarcopenia by a hand-grip strength < 20 kg for women and < 30 kg for men	all-risk mortality	Sarcopenic obesity men compared to control subjects in the presence of previous cardiovascular disease	HR: 1.47, 95% CI: 1.30–1.66)
Meta-analysis of 23 studies (18)	50 866	Varying according to each included study	all-risk mortality	Varying according to each included study	pooled HR = 1.21, 95% CI: 1.10–1.32, *p* < 0.001, *I* ^2^ = 64.3%

BMI, body mass index; CI, confidence interval; HR, hazard ratio; MAMC (Baseline midarm muscle circumference); WC, waist circumference.

Altogether, there is a core body of evidence that suggests that sarcopenic obesity must be considered a predictor of all-cause mortality among adults, thus justifying the implementation of a more systematic screening as well as preventive and therapeutic strategies that could decrease the mortality associated with this condition.

## 7 Management of sarcopenic obesity

The optimal therapeutic approach for sarcopenic obesity remains to be determined owing to the limited number of clinical trials conducted in this specific context.

**Figure 2:**
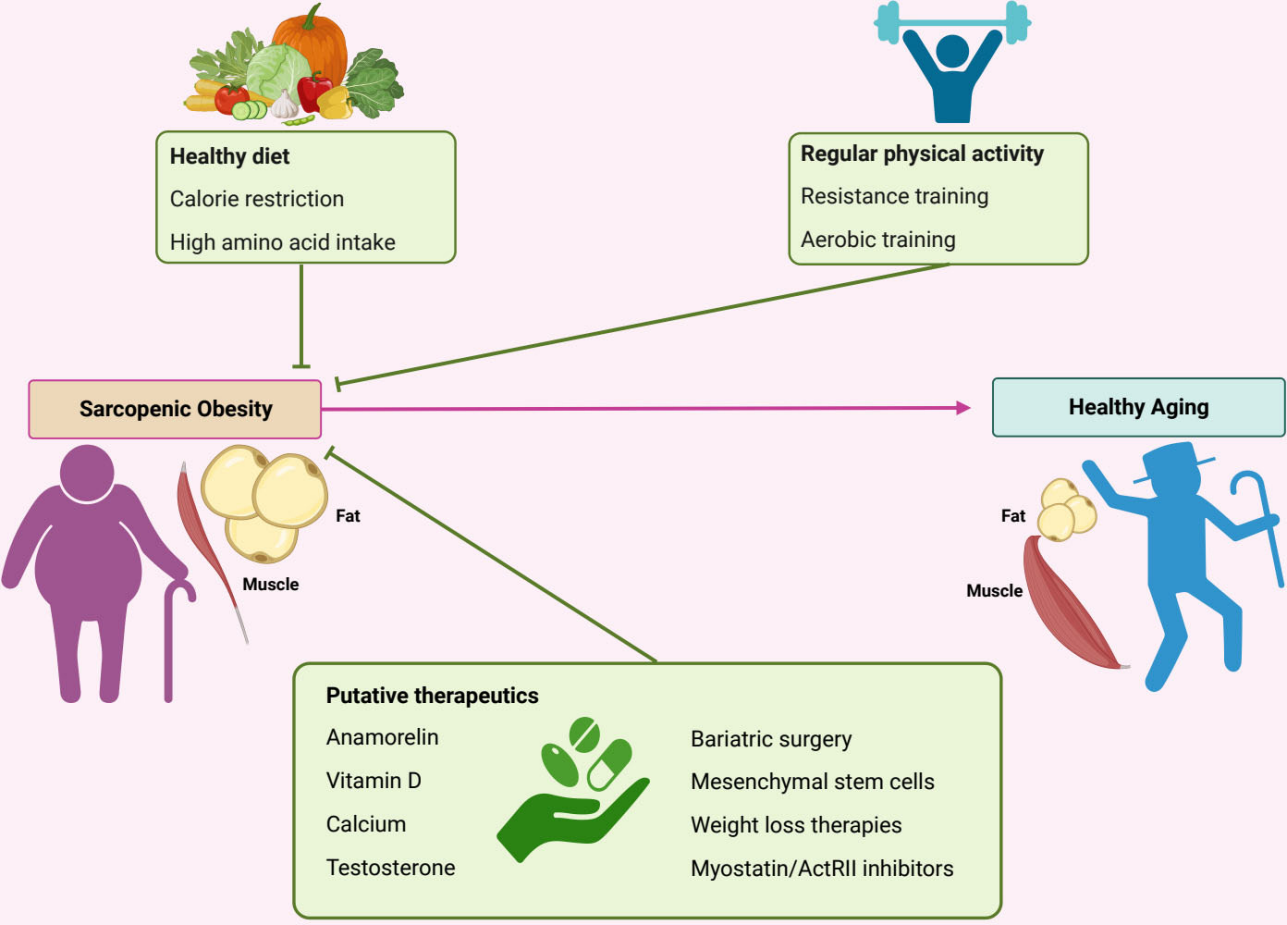
Therapeutic strategies to counter-act sarcopenic development. Various therapeutic approaches are proposed against sarcopenic obesity. Caloric restriction could be considered cautiously, especially in elderly subjects associated with high-quality protein intake. Physical activity is a cornerstone in the management of sarcopenic obesity and should combine aerobic and resistance exercises. Various pharmacological treatments are considered and include myostatin inhibitors, anamorelin, vitamin D, testosterone and selective androgen receptor modulators, and weight loss therapies. ActRII, Activin type II receptor.

Lifestyle approaches such as caloric restriction and physical exercise are considered the cornerstone of sarcopenic obesity treatment (**Figure 2**). With regarding to nutrition, an ideal approach has yet to be established. Indeed, weight loss in older obese individuals remains controversial, as it is a double-edged strategy that exerts a beneficial impact with decreased obesity-related complications and potential negative effects. Presently, a very-low calorie intake must be avoided in older individuals with sarcopenic obesity, as this strategy can compromise overall health by inducing micronutrient and electrolyte deficiencies, have a deleterious impact on skeletal muscle mass, and reduce bone mineral density (33). Although the precise quantity of kcal per day has not yet been defined, it must be less than 750 kcal per day (70). High-quality protein intake (1–1.2 g/kg/day), particularly those including sources of leucine, is generally recommended and can be employed concomitantly with a calorie restricted diet (71). However, caution is required while consuming high-protein diets because of to the risk of impairment of renal function. Medical and dietary management are important to establish a food program that allows moderate calorie restriction while optimizing protein intake (72).

Physical exercise is an efficient strategy for counteracting several pathophysiological aspects of both sarcopenia and obesity by promoting mitochondrial biogenesis, reducing low-grade inflammation, and decreasing insulin resistance and skeletal muscle cell apoptosis (73). Aerobic and resistance exercises are safe for individuals at the risk of falling (74).

Aerobic exercise improves cardiovascular function, reduces insulin resistance, enhances skeletal muscle capacity, and reduces mortality in older people (75, 76). Sporting activities are considered to be aerobic exercise and include walking, running, swimming, cycling, and rowing. Aerobic training is an effective approach for weight control and improving muscle function and mass in patients with sarcopenic obesity. However, few clinical studies have assessed the effects of aerobic exercise on sarcopenic obesity.

Resistance exercise is considered the most effective form of physical training for older people for inducing muscle hypertrophy, enhancing muscle function, and promoting weight loss (77). It is defined as an exercise that induces muscle contraction in response to external resistance, and encompasses activities such as squats, sit-ups, and planks. Clinical studies evaluating the impact of resistance exercise are often short-term studies having small sample sizes and often comprise individuals with either sarcopenia or obesity but rarely comprise individuals identified as specifically having sarcopenic obesity. However, it is likely that a significant proportion of those included do in fact meet the criteria for sarcopenic obesity. Current evidence indicates that resistance exercise improves muscle strength and body composition by increasing lean body mass (78, 79).

Sedentary behavior may also trigger the development of sarcopenic obesity by being either a primary contributing factor or a consequence of both obesity and sarcopenia. Indeed, physical capacity decreases with age, showing a more rapid decline in case of sedentary lifestyles. A study conducted in the UK including 1286 men aged over 70 years showed an association between sedentary time and a significant risk of sarcopenic obesity, independent of the level of moderate to vigorous physical activity (80). Another study conducted in adults of both sexes aged 18 years also observed an association between the presence of sarcopenic obesity and lack of physical activity assessed by the number of daily steps (81). Although there is a relatively abundant literature on the beneficial role of physical activity in the fight against sarcopenic obesity, the effect of a sedentary lifestyle on sarcopenic obesity remains relatively unclear. At the pathophysiological level, it can be hypothesized that the absence of physical activity allows various pathological phenomena to persist, such as insulin resistance, low-grade inflammation, expansion of adipose tissue, and loss of function and mass of the muscle tissue. Current evidence indicates an association between a sedentary lifestyle and sarcopenic obesity, which remains to be confirmed in large-scale prospective studies.

The approach to exercise prescriptions for sarcopenic obesity must be individualized. A program that includes a combination of resistance and aerobic activities may be more beneficial than either intervention alone (82). For aerobic exercise, approximately 65% of the peak heart rate should be reached, with the aim of reaching 75% of the peak heart rate during the exercise. Resistance training has to be focus on only one to two muscle groups, with the initial 8–12 repetitions at approximately 65% of the maximal level of force that the individual could produce in a single repetition. Progression should be aimed at using 2 to 3 muscle groups and 75% of the maximum intensity (33).

Supplementation with calcium and vitamin D is another important potential benefit in the management of sarcopenic obesity. In terms of pathophysiology, vitamin D represents an attractive approach as the binding of the vitamin D receptor to its ligand 1,25 (OH)2D3 triggers gene transcription associated with muscle function (83). Furthermore, preclinical data suggest that vitamin D exert a positive impact on obesity by improving mitochondrial function and reducing oxidative stress and low-grade inflammation. Although vitamin D deficiency appears to be associated with the presence of sarcopenic obesity, the effect of vitamin D supplementation in this population of individuals has not produced consistent or positive results (84, 85). Therefore, the role of vitamin D supplementation in the management of sarcopenic obesity requires further characterization. The only indirect recommendation regarding calcium and vitamin D supplementation is from the American Academy of Geriatrics, which recommends 1,000 IU of vitamin D3 per day with calcium in the older non-hospitalized population aged≥ 65 years to maintain serum vitamin D levels at 30 ng/ml (86).

Several other pharmacological treatments, including testosterone administration are emerging candidates for the treatment of sarcopenic obesity men. Although testosterone supplementation presents a clear physiological rationale, particularly for increasing muscle strength and function, the available data regarding the impact of testosterone administration on muscle function and mass remain limited and conflicting (87, 88). Currently, various scientific societies such as the Obesity Society or the American Association of Clinical Endocrinologists, do not recommend the administration of testosterone in the management of obesity and/or sarcopenia (89, 90).

Selective androgen receptor modulators (SARMs) present the advantage of selectively activating androgen receptors in the bones and muscles without androgenic induction in the rest of the body. This approach appears to be more beneficial than strength improvement for individuals requiring muscle mass gain, and it is expected that an effective transdermal SARM will become available in the near future (91).

Weight-loss therapies such as those using GLP-1 receptor agonists (RA) have shown to be beneficial for sarcopenic obesity, however the data are mainly produced in preclinical studies and proper specific parameters of sarcopenic obesity assessed before and after the introduction of GLP-1 RA are required (92). Bariatric surgery induced weight-loss by a malabsorptive and/or restrictive mechanism with a consistent effect on adipose tissue mass, but its benefit for skeletal muscle remain to be determined (93)

Myostatin is secreted by the myocytes and inhibits their differentiation and proliferation. Its level increased in individuals with sarcopenia and/or obesity. Activin are mainly secreted by gonads have been showed also to promote muscle wasting. Myostatin and Activin receptor type 2 (ActRII) inhibitors have been developed and shown to increase muscle mass and strength and as well as improve insulin sensitivity *in vitro* (94, 95). These molecules down-regulates the expression of myostatin in muscle and adipose tissues and therefore it may be highly relevant in the treatment of sarcopenic obesity.

Whole-body vibration therapy stimulates muscle contraction. It can increase muscle function to a level similar to that of classical resistance training, but with the advantage of being more convenient and safer. Although it could represent a promising treatment, its implementation in routine management requires further assessment because the available evidence remains limited, particularly for the treatment of sarcopenic obesity (96).

Mesenchymal stem cells represent an attractive future potential therapy for sarcopenic obesity because of their immunomodulatory qualities and multipotent nature; however, current data supporting their benefits remain limited, and future clinical studies are required to properly understand their mechanism of action in the specific context of sarcopenic obesity (97).

Anamorelin, an oral ghrelin receptor agonist that stimulates and enhances lean mass, has been proposed to counteract cancer cachexia and is considered for sarcopenic obesity, owing to its anabolic and anti-inflammatory properties. Currently available data are derived from its impact on cancer cachexia, demonstrating an improvement in lean mass; however, its effect on muscle function remains unclear (98).

Overall, the best therapeutic approach for sarcopenic obesity with the current most effective and largest evidence is lifestyle modification, including regular combined aerobic and resistance exercise with diet modifications that should include caloric restriction with the aim of reducing fat mass and increasing muscle mass and function to improve the quality of life and reduce mortality. Various pharmacological molecules have been considered; however, they are not yet strongly recommended because of the lack of strong evidence. Longer studies assessing the effects of a multimodal approach against sarcopenic obesity, CV events, and mortality will be of great interest.

## 8 Conclusion

In conclusion, the incidence of sarcopenic obesity is increasing, mainly because of the ever-increasing aging global population. It is a severe disorder associated with frailty, risk of falls, bone fractures, cardiovascular diseases, reduced independence, elevated morbidity, and increased hospitalization and mortality rates. Sarcopenic obesity is associated with a higher risk of cardiovascular events than those associated with sarcopenia or obesity individually. Therefore, it must be considered a significant public health issue. Its pathogenesis is multi-factorial and involves various metabolic, inflammatory, and, in particular hormonal aspects.

The lack of a universally used diagnostic method and definition criteria prevents the clear estimation of prevalence, which currently remains underestimated. Therefore, The diagnosis of sarcopenic obesity represents the initial challenge.

The early identification of this condition remains important, and adapted interventions should be considered to reduce its prevalence and associated deleterious outcomes. Novel therapeutic strategies are required to improve the poor prognosis.

## Author contributions

KG and DR conceived of the review and supervised the project. SW, TN, YZ, DR and KG wrote the manuscript. All authors contributed to the article and approved the submitted version.
